# Growth phenotype analysis of heme synthetic enzymes in a halophilic archaeon, *Haloferax volcanii*

**DOI:** 10.1371/journal.pone.0189913

**Published:** 2017-12-28

**Authors:** Naoki Kosugi, Takuma Araki, Junpei Fujita, Satoru Tanaka, Taketomo Fujiwara

**Affiliations:** 1 Department of Science, Graduate School of Integrated Science and Technology, Shizuoka University, Shizuoka, Japan; 2 Department of Environment and Energy Systems, Graduate School of Science and Technology, Shizuoka University, Shizuoka, Japan; 3 Department of Biological Sciences, Faculty of Science, Shizuoka University, Shizuoka, Japan; Universidade Nova de Lisboa Instituto de Tecnologia Quimica e Biologica, PORTUGAL

## Abstract

Halophilic euryarchaea lack many of the genes necessary for the protoporphyrin-dependent heme biosynthesis pathway previously identified in animals and plants. Bioinformatic analysis suggested the presence of two heme biosynthetic processes, an Fe-coproporphyrinogen III (coproheme) decarboxylase (ChdC) pathway and an alternative heme biosynthesis (Ahb) pathway, in *Haloferax volcanii*. PitA is specific to the halophilic archaea and has a unique molecular structure in which the ChdC domain is joined to the antibiotics biosynthesis monooxygenase (ABM)-like domain by a histidine-rich linker sequence. The *pitA* gene deletion variant of *H*. *volcanii* showed a phenotype with a significant reduction of aerobic growth. Addition of a protoheme complemented the phenotype, supporting the assumption that PitA participates in the aerobic heme biosynthesis. Deletion of the *ahbD* gene caused a significant reduction of only anaerobic growth by denitrification or dimethylsulfoxide (DMSO) respiration, and the growth was also complemented by addition of a protoheme. The experimental results suggest that the two heme biosynthesis pathways are utilized selectively under aerobic and anaerobic conditions in *H*. *volcanii*. The molecular structure and physiological function of PitA are also discussed on the basis of the limited proteolysis and sequence analysis.

## Introduction

Heme plays an important biochemical role as a coenzyme of the proteins catalyzing various redox reactions [1]. In all the eukaryotes and some prokaryotic microbes, protoporphyrin IX is synthesized by six successive reactions from aminolevulinic acid as the starting material until finally protoheme is generated by insertion of a ferrous ion into the protoporphyrin by ferrochelatase (PpfC) [2, 3]. Recently, two biosynthetic pathways, of which the biochemical processes differ from that of the protoporphyrin-dependent pathway, have been newly found in some bacteria and archaea (S1 Fig). An alternative heme biosynthesis (Ahb) pathway has been identified in anaerobic microbes including anaerobic sulfate- or nitrate-respiring bacteria and methanogenic archaea [4, 5]. In the Ahb pathway, siroheme, the intermediate compound generated from uroporphyrinogen III, is converted to protoheme through oxidative decarboxylating reactions [4, 6]. The protoporphyrin-dependent pathway proceeds aerobically, and oxygen molecules (O_2_) are used for oxidative decarboxylation of the propionyl bases of the intermediate compounds [2]. In contrast, all the reaction steps of the Ahb pathway proceed under anaerobic conditions, and the radical SAM enzymes catalyze the oxidative decarboxylation of the intermediates to yield protoheme [4–6].

More recently, Dailey and co-workers have reported the presence of a novel heme biosynthetic pathway in the gram-positive bacteria in the phyla Firmicutes and Actinobacteria [3, 7, 8]. In this aerobic pathway, Fe-coproporphyrin III (coproheme) decarboxylase (ChdC, formerly designated HemQ) catalyzes the oxidative decarboxylation of coproheme and yields protoheme using hydrogen peroxide, not O_2_, as a putative oxidant [9–11]. Crystal structures of ChdC from *Geobacillus stearothermophilus* and *Listeria monocytogenes* have demonstrated the homopentameric architecture and putative reaction mechanism of the enzyme [11, 12].

Blast searching reveals that the functional genes of the novel heme biosynthetic pathways are widely distributed in bacteria and archaea [8]. *Haloferax volcanii* is an extremely halophilic microorganism that belongs to the phylum euryarchaeota, class halobacterium, and occupies an ecological niche in hypersaline environments such as salt lakes and salt ponds [13, 14]. Bioinformatic analysis reveals that there are no genes in the *H*. *volcanii* genome for successive catalytic reactions to synthesize protoheme from uroporphyrinogen through the protoporphyrin-dependent pathway, except for the *pgdH1* gene encoding anaerobic protoporphyrinogen dehydrogenase [15]. On the other hand, similar to another halophilic archaeon, the functional genes related to the Ahb pathway, *nirDL* and *nirGH* (both homologous to *ahbAB*), *ahbC*, and *ahbD*, have been identified in the *H*. *volcanii* genome [3, 4, 16, 17] (S1 Fig).

In addition, a putative *chdC* gene encoding a protein that may catalyze the oxidative decarboxylation of a coproheme as the final step of the aerobic ChdC pathway, formerly known as the HemQ pathway, has been identified in the *H*. *volcanii* genome [3, 8]. This protein, which was first reported by Bab-Dinitz *et al*. [18] and designated ‘PitA’, is assumed to be a soluble cytoplasm-localized protein that is 501 amino acids in length, being the N-terminal region of 250 amino acid residues homologous to ChdC. A distinctive feature of PitA is that an antibiotic biosynthesis monooxygenase (ABM)-like domain with 200 amino acid residues is fused on the C-terminal side of the putative ChdC domain by a histidine-rich linker sequence [18]. The *H*. *volcanii* PitA was purified by Co^2+^-affinity, due to the binding affinity of its histidine-rich linker region to this divalent metal [18]. A *pitA* gene with similar structural characteristics is present in all haloarchaeal species whose total genome information is already available. However, involvement of the PitA in heme biosynthesis has not been confirmed in *H*. *volcanii* and other halophilic archaea.

In this study, *pitA* and *ahbD* gene deletion variants of *H*. *volcanii* were constructed, and their growth phenotypes were analyzed. The *pitA* deletion (Δ*pitA*) variant grew very slowly under aerobic conditions, while it grew anaerobically by denitrification or dimethylsulfoxide (DMSO) respiration with a growth rate similar to that of the parent strain. Aerobic growth of the Δ*pitA* variant was restored by addition of protoheme to the medium. On the other hand, the Δ*ahbD* variant grew in the same manner as the parent strain under aerobic conditions but not by denitrification or DMSO respiration under anaerobic growth conditions. Anaerobic growth of the variant was recovered by supplementation with protoheme. The results of the growth phenotype analysis are consistent with the assumption that the *pitA* and *ahbD* genes are involved in heme biosynthesis by the aerobic ChdC pathway and anaerobic Ahb pathway of *H*. *volcanii*. Limited proteolytic digestion and sequence analysis were also performed for characterization of the unique molecular structure and physiological function of PitA.

## Materials and methods

### Strains and growth conditions

*H*. *volcanii* strain H26, an orotate:phosphoribosyl transferase (PyrE2) mutant was kindly supplied by Dr. T. Allers (Inst. Genetics, Nottingham Univ., UK) and used for experiments of cultivation, disruption of the *pitA* and *ahbD* genes, growth genotype analysis, and purification of PitA [19]. Cultivation of *H*. *volcanii* was carried out using Hv medium prepared according to Hattori *et al*. [20]. Small scale cultivation was performed as follows: a cultivation tube, 20 mL in volume and containing 3 mL Hv medium, was inoculated with frozen stock of the archaeal cells. For aerobic cultivation, the cultivation tubes were covered by an aluminum cap and shaken at 120 rpm at 37°C in the dark. Growth was monitored by measuring the OD_600_ using a spectrophotometer (mini photo 518R, Taitec Co., Saitama, Japan). When OD_600_ reached 0.6–0.8 (mid-exponential), cells were collected and used for gene disruption experiments or as inoculants for cultivation experiments.

Analysis of the growth phenotype under aerobic conditions was carried out by measuring the OD_600_ every 24 h after inoculation of the preculture. Anaerobic cultivation was carried out according to Hattori *et al*. [20] as follows. The medium (3 mL) containing 50 mM KNO_3_ or 50 mM DMSO as the respiratory substrate for denitrification or DMSO respiration, respectively, was placed in the cultivation tubes. The tubes were sealed with butyl rubber, then the gas phase in the tube was exchanged by gentle bubbling with N_2_:O_2_ (99.8:0.2 [vol/vol]) mixed gas (Shizuoka Sanso Co., Shizuoka, Japan) for 5 min using a sterile needle. The tubes were shaken at 80 rpm at 37°C. The gas phase in the tube was purged by the same mixed gas every 24 h. The effects of protoheme on growth of the strains was examined in the above-mentioned media, which were supplemented by protoheme to reach 5 μM as the final concentration. Protoheme was prepared from hemoglobin (Nacalai Tesque Inc., Kyoto, Japan) by the method of acetone-HCl extraction [21]. The extracted material was analyzed using HPLC system (LC-30AD Shimadzu, Kyoto, Japan) equipped with an octadecyl silica column (Mightysil RP-18 GP 250–4.6 (5 μm), Kanto Chemical Co., Inc. Tokyo, Japan). The sample adsorbed on the column was eluted at a flow rate of 0.5 mL min^-1^ at 30°C using a linear gradient generated from 0.1% tetrafluoroacetate (TFA)/distilled water and 0.1% TFA/acetonitrile. A single peak appeared on the chromatogram monitoring the absorbance of the effluent at 400 nm. Eluted pigment was identified spectrophotometrically using a photodiode array detector (SPD-M30AD; Shimadzu). The concentration of protoheme was quantified spectrophotometrically using the ε_557_ = 34.4 mM^-1^cm^-1^ of pyridine ferroprotohemochome [1]. Stock solutions of protoheme for supplementation of the growth media were prepared by dissolving concentrated protoheme in pure DMSO (to a final concentration of 2 mM) or in 1 M Tris-HCl buffer (pH 8.0) (final concentration of 1.2–1.4 mM).

### Construction of *pitA* and *ahbD* gene deletion variants

A uracil synthase-deficient mutant of *H*. *volcanii*, strain H26 (Δ*pyrE2*), was used for gene disruption experiments by a pop-in/pop-out method based on uracil auxotrophy [19]. The standard protocols used for DNA handling in *E*. *coli* and *H*. *volcanii* followed Sambrook and Russell [22] and Dyall-Smith [23], respectively. A 2.7 kbp fragment, including upstream (0.5 kbp) and downstream (0.7 kbp) regions of the *pitA* gene, was amplified using a set of pitAUF/pitADR oligonucleotide primers. Sequences of the primers are shown in S1 Table. PCR amplification was carried out using KOD-plus DNA polymerase (Toyobo Co., Ltd., Osaka, Japan). The amplified fragment was cloned into a pCR-blunt II TOPO vector (Life Technologies, Carlsbad, CA), yielding pCR*pitA*. The DNA sequence of the PCR product was determined using a SequeCEQ 8000 Genetic Analysis System (Beckman Coulter, Inc., Brea, CA). Next, an inverse-PCR amplification was carried out using the pCRpitA plasmid as the template and a set of pitADF/pitAUR primers to remove an internal region (0.7 kbp) of the *pitA* gene. The product was purified, treated by the restriction enzyme *Bam*HI (Takara), then subjected to self-ligation (Ligation High ver. 2, Toyobo), yielding pCRΔ*pitA*. The inserted fragment of 2.0 kbp nucleotides was extracted by *Sac*I/*Pst*I double digestion and cloned into the same restriction site of pTA131, an integration vector containing *pyrE2* gene (supplied by Dr. T. Allers), generating pΔ*pitA*. After demethylation for an effective transformation, the pΔ*pitA* plasmid was introduced into *H*. *volcanii* strain H26 for integration into the genome DNA [24]. Colonies of the pop-in strain, designated P01 (genotype: Δ*pyrE2 pitA*^+^ [*pyrE2* Δ*pitA*]), the pΔ*pitA* plasmid integrated on the chromosome of the strain H26 is indicated by brackets), appeared on the uracil-deficient casamino (Hv-Ca) agar medium because uracil prototrophy was restored by homologous recombination of the plasmid [19].

Strain P01 was pre-cultivated under aerobic and anaerobic (denitrifying) conditions in the Hv-Ca medium. Aerobically cultivated P01 cells were streaked on the Hv-Ca agar medium supplemented with 50 μM 5’-FOA and 50 μM uracil for a second homologous recombination event. The agar medium was incubated at 37°C. Due to the toxicity of 5’-fluorouracil, which is a catalytic product of 5’-fluoroorotic acid (5’-FOA) by PyrE2, only the pop-out strain formed colonies on the agar medium. Cultivation of *H*. *volcanii* strain P01 under denitrifying condition was also performed using Hv-Ca medium supplemented with 50 mM KNO_3_. Denitrifying cells of strain P01 thus obtained were streaked on Hv-Ca agar medium that was supplemented with 50 μM 5’-FOA, 50 μM uracil, and 5 mM NaNO_3_. The resulting agar medium was placed into an anaerobic container with an oxygen absorber (AnaeroPack, Mitsubishi Gas Chemical Co., Inc., Tokyo, Japan). Preparation of the genomic DNA from each colony was carried out according to Dyall-Smith [23] with slight modifications. Destruction of the *pitA* gene was confirmed by PCR amplification of the corresponding region of the chromosomal DNA. The deletion variant of the *pitA* gene (genotype: Δ*pitA* Δ*pyrE2*) obtained was designated P02.

The *ahbD* gene deletion variant, designated strainA02 (genotype: Δ*ahbD* Δ*pyrE2*), was also prepared according to an experimental procedure that was similar to that of the *pitA* gene deletion variant as described in the supporting information.

### Characterization of molecular properties of PitA

PitA was purified from *H*. *volcanii* H26 cells cultivated under aerobic or denitrifying conditions according to Bab-Dinitz *et al*. [18] with some modifications as mentioned in the supporting information. Relative molecular mass (*M*_r_) of the subunit protein of PitA was determined by SDS-PAGE according to the method of Schägger and von Jagow [25]. The protein concentration was measured using a BCA protein assay kit (Pierce, Rockford, IL, USA) with bovine serum albumin (BSA) as the standard. Spectroscopic analysis in the visible/near ultraviolet region was carried out in a 1 cm light-path cuvette using an MPS-2000 spectrophotometer (Shimadzu, Kyoto, Japan). The *M*_r_ of PitA in the solution was estimated based on the elution profile by Sephacryl S-300 (Pharmacia Biotech AB, Uppsala, Sweden) gel filtration using thyroglobulin (*M*_r_ = 669,000), ferritin (440,000), *Haloarcula marismortui* KatG catalase-peroxidase (160,000), BSA (66,000), *Nitrosococcus oceani* cytochrome *c*_554_ (25,000), and horse mitochondrial cytochrome *c* (12,500) as standard proteins [26, 27].

### Proteolytic digestion of PitA

The purified PitA (100 μg protein) was dissolved in 100 μL of 20 mM Tris-HCl buffer (pH 8.0) containing 2 M NaCl and 10 mM CaCl_2_. Proteolytic digestion was started by adding proteinase K (Wako Pure Chemical Industries, Ltd., Osaka, Japan) to the PitA solution to reach 10 ng/μL, then the resulting solution was incubated at 37°C. After appropriate reaction times, a small amount of the solution was sampled, treated with 4% each of SDS and β-mercaptoethanol, and then analyzed by SDS-PAGE. The protein bands on the polyacrylamide gel were electrophoretically transferred to polyvinylidene difluoride membrane (Millipore) using an electroblotting apparatus (Atto Co., Tokyo, Japan), then the N-terminal sequence was determined by subjecting the blotted membrane to the protein sequencer model PPSQ-21A (Shimadzu). Fractionation of the proteolytic fragments of PitA by proteinase K was also carried out as follows. After 1 h incubation, the proteolysis was stopped by adding 10 μM phenylmethylsulfonyl fluoride (PMSF) to the solution. The resulting sample was loaded on a column (2 cm × 120 cm) of Sephacryl S-200 (Pharmacia) that had been equilibrated by 20 mM Tris-HCl buffer (pH 8.0) containing 2 M NaCl and 10 μM PMSF. Apparent *M*_r_s of each fractions were also estimated as described above.

## Results and discussion

### Preparation and phenotypic analysis of Δ*pitA* variant

The *pitA* gene was removed by the double-integration method utilizing the uracil synthase-deficient strain H26 (genotype: Δ*pyrE2*). The pop-in strain P01, obtained by integration of the gene destruction plasmid pΔ*pitA* into the chromosome of the strain H26, was cultivated on agar medium supplemented by 5’-FOA and KNO_3_ under aerobic or anaerobic (denitrifying) conditions for the *pitA* gene pop-out, as described in the Methods. Sixty-four and 54 colonies were collected from aerobic and denitrifying agar media, respectively, then the absence of the *pitA* gene was confirmed by PCR amplification of the corresponding region of the gene. Destruction of the *pitA* gene was confirmed in one colony each obtained from aerobic and denitrifying agar media, respectively, while the remaining 116 strains were shown to be revertants. The former colony was subcultured under aerobic conditions, but it was finally replaced by the revertant, probably due contamination by revertant cells. It has been reported that the *pitA* gene destruction by the similar pop-in/pop-out technique was tried but not been achieved, and the result has been explained by the essential role of PitA [28]. In this study, we have succeeded in obtaining the *pit*A gene deletion mutant by performing the pop-out procedure under denitrifying (anaerobic) conditions. Isolation of the *pitA* deletion variant was accomplished by subcultivation of the latter colony under denitrifying conditions; then, the strain P02 (genotype: Δ*pyrE2* Δ*pitA*) obtained was used for experiments as the Δ*pitA* variant of *H*. *volcanii* (panel A in S2 Fig).

**Fig 1:**
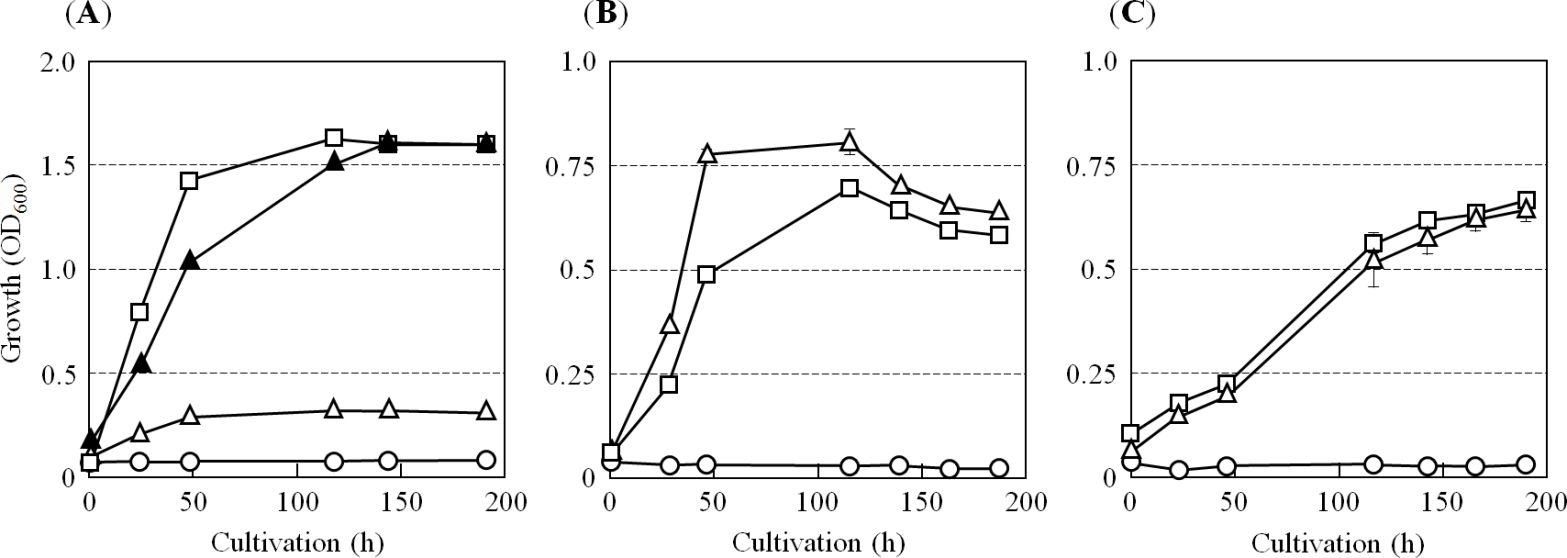
Aerobic growth variant phenotype of the Δ*pitA* variant of *H*. *volcanii*. The *pitA* deletion variant of *H*. *volcanii*, strain P02 (open triangles), and the parent strain H26 (open squares) were cultivated under aerobic or anaerobic conditions. As indicated in (**A**), the aerobic growth of strain P02 was strongly suppressed, and the OD_600_ value reached a maximum of only approximately 0.3. Aerobic growth of strain P02 was restored to almost the same level of that of strain H26 by supplementation of a protoheme stock solution (dissolved in pure DMSO) to reach 5 μM as the final concentration to the medium (closed triangles). The OD_600_ of the medium without inoculation of the archaeal cells is indicated by open circles in the figures. Under anaerobic condition, strain P02 grew actively by denitrification (**B**) or by DMSO-respiration (**C**). Experiments were performed independently three times. Error bars represent standard error (S.E.). The S.E. values were small, and therefore the error bars are sometimes masked by the symbols.

Under aerobic conditions, the parental strain H26 grew actively and the optical density at 600 nm (OD_600_) of the culture reached 1.6 in the stationary phase after cultivation for 4–5 days. The growth rate of strain P02 was greatly reduced, and the OD_600_ increased only to 0.3 in the stationary phase (Fig 1A). In contrast, strain P02 grew at almost the same rate as the parental strain under denitrifying conditions (Fig 1B). Anaerobic growth by DMSO-respiration of *H*. *volcanii* was also not affected by destruction of the *pitA* gene (Fig 1C). Consistent with the above-mentioned difficulty in preparation of the Δ*pitA* variant, the results demonstrate that, although deletion of the *pitA* gene is not lethal, PitA may play some significant roles in the aerobic growth of *H*. *volcanii*, but not in growth by anaerobic respiration. Our result also agrees with the previous report that deletion of the *chdC* gene in *Staphylococcus aureus* gives a small colony variant phenotype that is caused by a slow growth under aerobic conditions [29].

Many proteins that contain the protoheme and/or its derivatives, heme *a* and heme *c*, as prosthetic cofactors are involved and function significantly in the metabolisms, especially in the respiratory processes, as redox enzymes and electron carriers [1]. PitA has been expected to be involved in an aerobic heme biosynthesis in the halophilic archaea by the ChdC pathway [8]. Therefore, if the repression of aerobic viability observed in the *pitA* deletion variant is due to the loss of heme synthesis, aerobic growth would be recovered by artificial supplementation of protoheme. As expected, strain P02 grew aerobically in the medium containing 5 μM protoheme at a rate similar to that of the parent strain H26 (Fig 1A).

### Preparation and phenotypic analysis of the Δ*ahbD* variant

The second heme biosynthesis process in *H*. *volcanii*, the Ahb pathway, is expected to function under an anaerobic condition [4–6]. Therefore, in contrast to the results of phenotypic analysis of the *pitA* deletion variant, it is anticipated that only the anaerobic growth of the archaea will be suppressed by deletion of the *ahbD* gene. The *ahbD* gene was also eliminated by a protocol similar to the preparation of the Δ*pitA* variant except for the pop-out procedure as described in the Supplementary materials. The pop-in strain A01, prepared by integration of the pΔ*ahbD* into the chromosomal DNA of *H*. *volcanii*, was cultivated on the 5’-FOA-containing agar medium under aerobic conditions. Two of three pop-out strains that appeared on the agar medium were confirmed to be the *ahbD* gene deletion variants and the remaining strain was a revertant. The *ahbD* gene deletion variant obtained was designated A02 (genotype: Δ*ahbD* Δ*pyrE2*) and used for the cultivation experiments.

**Fig 2:**
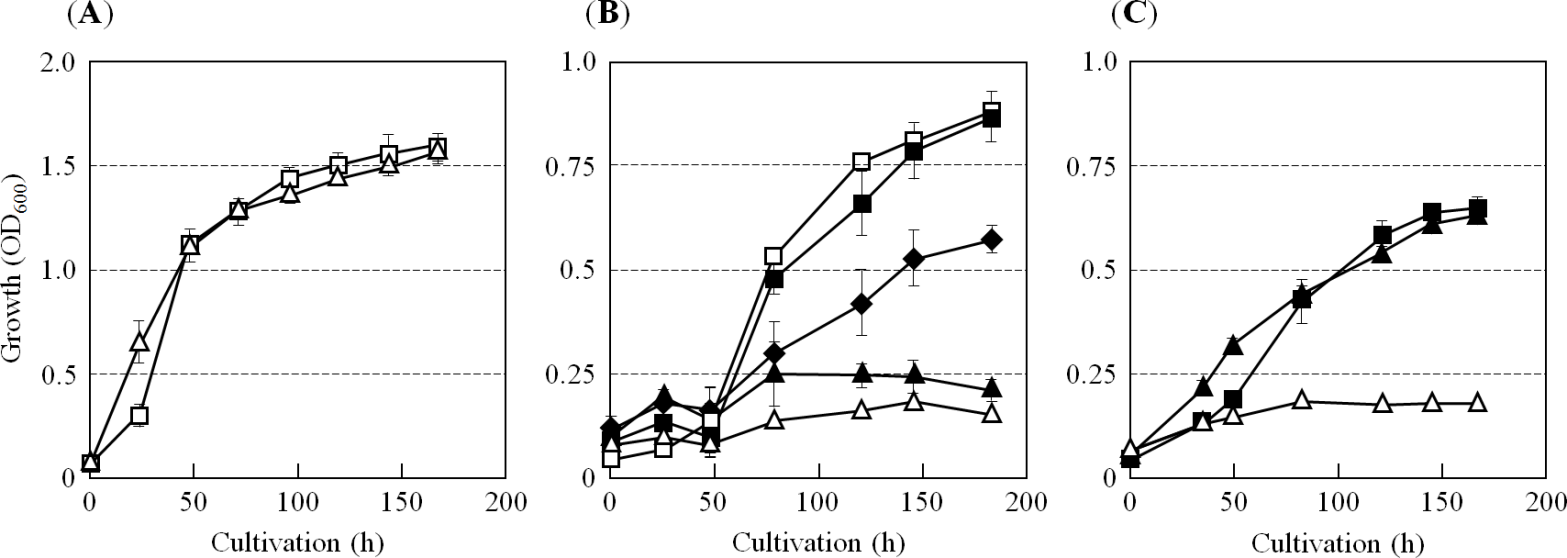
Anaerobic growth variant phenotype of the Δ*ahbD* variant of *H*. *volcanii*. The *ahbD* deletion variant of *H*. *volcanii*, strain A02 (open triangles), and the parent strain H26 (open squares) were cultivated under aerobic or anaerobic conditions. As shown in (**A**), strain A02 grew actively under aerobic conditions with a similar growth rate to that observed in strain H26. Under the denitrifying condition, growth of the strain A02 was strongly inhibited as shown in (**B**). Denitrifying growth of strain A02 was not recovered by adding protoheme up to 5 μM (closed triangles) using the stock solution of protoheme dissolved in the Tris-HCl buffer. The growth was restored in some extent by further addition of 1 mM DMSO to the medium (closed diamonds), the OD_600_ value reached approximately 0.6. Denitrifying growth of strain H26 was not affected by addition of 5 μM protoheme (DMSO was not added, closed squares). As indicated in (**C**), with supplementation of 5 μM protoheme, anaerobic growth of the strain A02 by DMSO-respiration (closed triangles) was restored to a similar level to that of strain H26 supplemented with protoheme (closed squares). Experiments were performed independently three times. Error bars represent S.E.

Strain A02 grew under aerobic conditions at a rate similar to that of the parent strain H26 (Fig 2A). As expected, strain A02 grew very slowly under anaerobic condition by denitrification and DMSO-respiration (Fig 2B and 2C). The denitrifying growth was not recovered by addition of 5 μM protoheme to the media by using a protoheme stock solution in which protoheme was dissolved in the Tris-HCl buffer (Fig 2B). However, when 1 mM DMSO was added to the medium in addition to 5 μM protoheme, the OD_600_ of strain A02 increased to 0.57 after 180 hours cultivation. DMSO is an amphipathic chemical that has been known to facilitate permeation of hydrophobic or hydrophilic compounds into cells [30]. The experimental result is explainable by the hypothesis that addition of DMSO was necessary for permeation of the protoheme across the archaeal cell membrane because the negative charge of the protoheme coordinated with chloride ions. Denitrifying growth of *H*. *volcanii* has been shown to be suppressed by DMSO in a concentration-dependent manner (Koyanagi *et al*. manuscript in preparation). Partial restoration of the denitrifying growth of strain A02 may due to the DMSO-dependent suppression of denitrification. The DMSO-respiring growth of the strain A02 was restored to a similar level to that of strain H26 by supplementation of 5 μM protoheme to the media (Fig 2C).

On the other hand, aerobic growth of the strain P02 (Δ*pitA* mutant) was recovered by addition of 5 μM protoheme to the media by using a protoheme/Tris stock solution, as shown in S3 Fig. Protoheme-dependent aerobic growth of the strain P02 without an addition of DMSO (11.4 h doubling time (t_d_)) was slow compared with that of coexistence with DMSO (7.6 h t_d_). The result can not be explained only by the amphipathic feature of DMSO. It has been reported that many microorganisms including not only pathogenic but also non-pathogenic bacteria possess ATP-binding cassette (ABC) transporter that participates in the active transport of heme across the membrane into the cell [31]. Many genes encoding ABC transpoter, including transporter of metal complexes such as siderophore, heme, and vitamin B12, are identified in the *H*. *volcanii* genome, while most of their physiological substrates have not been clarified yet [32]. Restoration of protoheme-dependent aerobic growth of strain P02 without addition of DMSO might be explained by the assumption of the presence of ABC heme transporter in *H*. *volcanii*. That is because an efficient energy-yielding by aerobic respiration will be able to supply ATP enough for an inward transport of heme by the transporter.

Denitrification-related genes, including dissimilatory nitrite reductase *nirK* (HVO_2141), nitric oxide reductase *norB* (HVO_2147), and blue copper-containing electron carrier proteins (HVO_2145 and HVO_2150), are present in the vicinity of the *ahbD* gene (HVO_2144) in the *H*. *volcanii* genome [15]. A palindromic sequence (CGAA-X_4_-TTCG), which is a potent recognition sequence of a helix-turn-helix type regulator, is commonly identified in the promoter sequence of the denitrification-related genes and of the *ahbD* gene. A reporter assay experiment reported by Hattori *et al*. [20] demonstrated that the *nirK* gene is activated under anaerobic conditions, and the conserved palindromic sequence is essential for the transcription activity. These results suggest that the *ahbD* gene would also be activated under anaerobic conditions, being consistent with the anaerobic induction of the Ahb pathway.

### Molecular characterization of PitA

**Fig 3:**
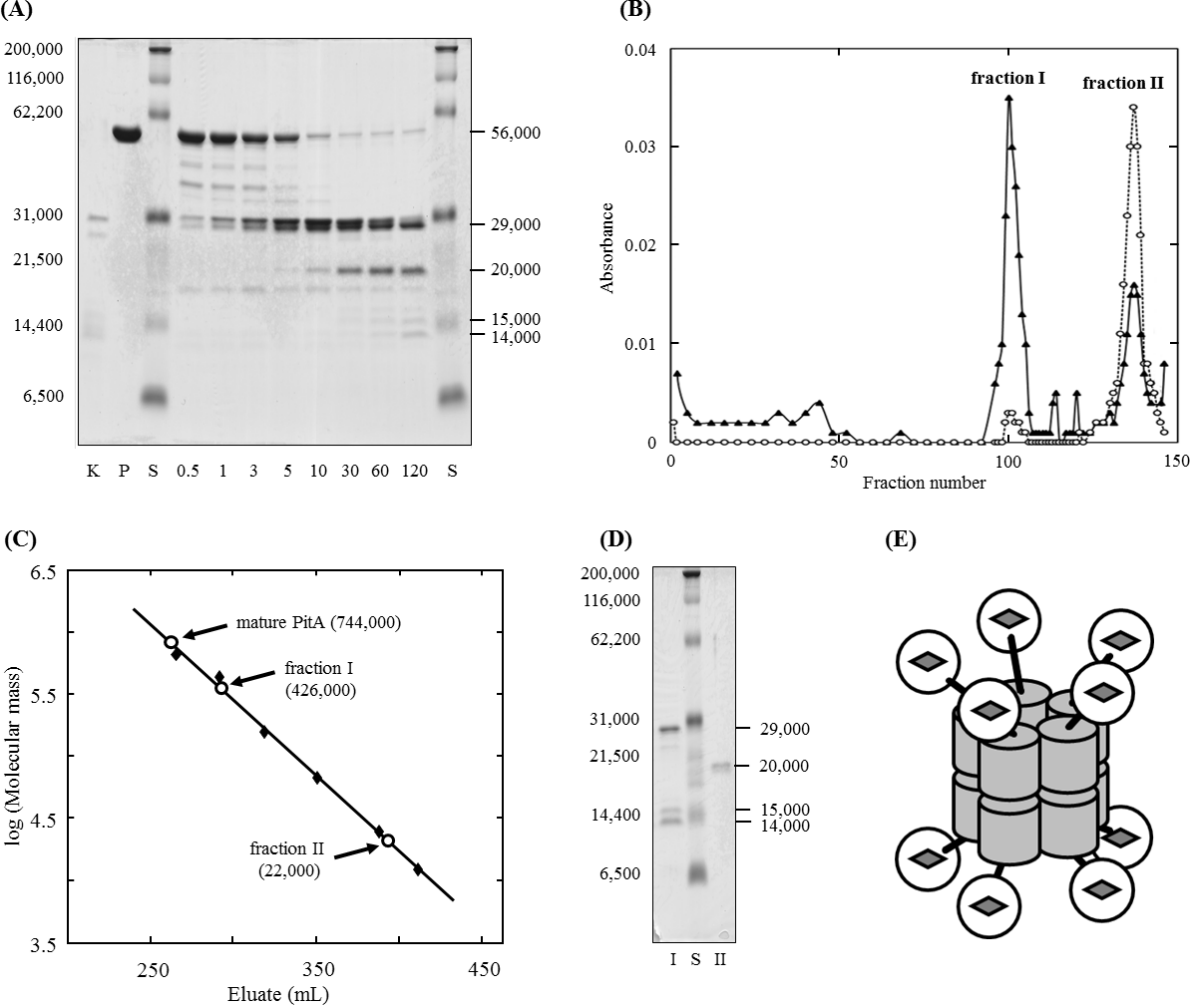
Molecular characterization of PitA by limited proteolytic analysis. SDS-PAGE of the proteinase K-digested fragments of PitA is shown in (**A**). Numbers below the lanes indicate each reaction times (min) after the start of proteolysis. In lanes P and K, the purified PitA and proteinase K used in the experiment, respectively, were loaded. The *M*_r_ standard was loaded in lane S. Estimated *M*_r_s of the fragments are indicated on the right side of the gel, while those of the standard proteins are on the left side. In (**B**), the proteolytic digest obtained after the 120 min reaction was fractionated by gel filtration. Proteins and heme moieties collected in each fractions were monitored by measuring the absorbance at 280 nm (closed triangles) and at 410 nm (open circles), respectively. Apparent *M*_r_s of the two fractions, I and II, that were eluted at around the 100^th^ and 147^th^ tubes, respectively, were estimated as shown in (**C**) using the *M*_r_ standard proteins as described in the Methods. Estimation of the *M*_r_ of PitA in the mature state was performed similarly. Results of the SDS-PAGE of the fractions I and II are indicated in (**D**). N-terminal amino acid sequences of each fragments were determined as described in the Results and Discussion. A possible structural model of the PitA estimated from these results is represented in (**E**).

PitA was purified from the soluble fraction of the *H*. *volcanii* cells by Ni^2+^-affinity, hydrogen-binding, and size-exclusion chromatographies. The purified PitA thus obtained was confirmed to be electrophoretically homogeneous by SDS-PAGE (Fig 3A). Thirty-one micrograms of the purified preparation was obtained from 1 g (wet weight) of *H*. *volcanii* cells. The purified preparation (17 μg protein/g of cells) was also obtained from the denitrifying cells of *H*. *volcanii*, indicating that expression of PitA occurs commonly regardless of whether the growth conditions are aerobic or anaerobic. The purified preparation contained protoheme with 14.9 nmol/mg protein as the prosthetic cofactor. The mole ratio of the protoheme of the subunit molecule whose *M*_r_ was 56,000 was estimated to be 0.83, suggesting a one-to-one stoichiometry of protoheme in the subunit molecule of PitA. Absorption spectra of the purified PitA of the oxidized form, reduced form, and reduced form in complex with carbon monoxide indicated that the heme moiety of PitA is in the five-coordinated, high-spin state (S4 Fig). The *M*_r_ of PitA in the solution was determined to be 774,000 by size-exclusion chromatography, suggesting a homomultimeric architecture composed of more than ten molecules of the subunit as described below.

In a limited proteolytic experiment, the subunit protein of PitA was digested into two main fragments whose apparent *M*_r_s were 29,000 and 20,000 after 120 minutes incubation with proteinase K, as shown in Fig 3A. The N-terminal amino acid sequences of the former fragment was determined to be VEAPQ, which is identical to the 2^nd^ to 5^th^ residues of the putative amino acid sequence of the PitA, while that of the latter fragment was determined to be AGKPH, which is identical to the 322^nd^ to 326^th^ residues. The results indicate that the digested fragments whose apparent *M*_r_s were 29,000 and 20,000, corresponding to the ChdC domain and the ABM-like domain of PitA, respectively. Some protein bands with the apparent *M*_r_s of 40,000–50,000, which appear temporarily in the early period of proteolysis, probably correspond to the ChdC domain and/or the ABM-like domain which is attached to the linker sequence. Intensity of the protein band with an apparent *M*_r_ of 19,000 increased at 3 minutes after starting proteolysis, and then decreased gradually, suggesting that it is also an proteolytic fragment that appears temporarily during degradation. Protein bands with apparent *M*_r_s of 15,000 and 14,000 appeared after 60 minutes incubation of PitA with proteinase K. N-terminal amino acid sequences of the former and latter fragments were determined to be VEAPQ and RYIEG, which are identical to the 2^nd^ to 5^th^ residues and 132^nd^ to 136^th^ residues of PitA, respectively, indicating that the two fragments were proteolytic cleavage products of the ChdC domain.

As shown in Fig 3B, a mixture of the proteolytic digests of the PitA obtained after 120 minutes incubation with proteinase K was fractionated by size-exclusion chromatography into two fractions, I and II. Fraction I was colorless, while fraction II displayed a pale red-brown color due to the coexistence of protoheme. The *M*_r_s of the protein molecule collected in fractions I and II were estimated to be 426,000 and 22,000, respectively (Fig 3C). SDS-PAGE analysis revealed that the ChdC domain and its two proteolytic fragments were collected in fraction I, while the ABM-like domain was collected in fraction II (Fig 3D). The results indicated that the ChdC domain exists in a multimeric state with a high *M*_r_ in the solution, while the ABM-like domain is in the monomeric state and contains protoheme.

To date, a ring-shaped pentameric structure has been reported according to the crystal analysis of ChdC from a gram-positive bacteria [9, 11]. A similar pentameric structure has also been determined in the TT1485 gene product from *Thermus thermophiles*, a putative ChdC homologous with chlorite dismutase [33]. On the other hand, a hexameric structure has been reported for the chlorite dismutase from *Azospira oryzae* strain GR-1 [34]. It is difficult to explain the large molecular size (estimated *M*_r_ = 774,000) of PitA based on the pentameric or hexameric configuration of the subunit molecule. Hence, a structural model of the *H*. *volcanii* PitA is represented in Fig 3E. In this model, the PitA molecule consists of two back-to-back rings of each pentamer. Formation of the multimeric structure of PitA may due to the binding interaction among the ChdC domains of each subunit molecule. The ABM-like domain bound with the ChdC domain via a histidine-rich flexible linker sequence, but it might not be involved in forming the multimeric configuration of PitA in the solution. The protoheme moiety is considered to be involved in the ABM-like domain, as judged from the proteolytic examination shown in Fig 3B. It is noted that, considering the potential inaccuracy of gel filtration, structural models of dodecamer (hexamer x 2) and tetradecamer (heptamer x 2) are also possible and should not be excluded. For elucidating the subunit structure of PitA, application of a more accurate method for the determination of the *M*_r_ is required.

### Functional implications of the ABM-like domain

Recombinant ChdCs from *S*. *aureus* and *L*. *monocytogenes* are purified as apoproteins not containing protoheme [9, 11]. Values of the apparent dissociation constant K_D_ with protoheme has been estimated in the range of 1 to 40 μM, being consistent with the absence of protoheme in the purified ChdC due to the low binding affinity [7, 11, 29]. In the purified PitA, the ratio of the protoheme was determined by the subunit molecule to be 0.83 (mol/mol), which approximately fits a stoichiometric trend, while the ChdC domain obtained by limited proteolysis did not contain protoheme, and the heme moiety was found to bind totally in the ABM-like domain. These results seem suggestive of the physiological function of the ABM-like domain of PitA. An ABM-like domain has been found in proteins that are involved in a wide range of biochemical processes such as metabolism and transcription, as well as biosynthesis of secondary metabolites [35, 36]. *S*. *aureus* IsdG is one the ABM-domain proteins possessing heme oxygenase activity under coexistence with oxygen and a suitable electron-donating substrate such as ascorbate [37]. Based on the crystal analysis of the IsdG and its homologous enzymes, the tertiary structure of the active site and amino acid residues essential for activity were elucidated [37–39]. Sequence alignment and comparison of the predicted secondary structures suggested that the His^444^ (*H*. *volcanii* PitA numbering) was a putative proximal ligand of the protoheme in the AMB-like domain (S5 Fig). However, other residues essential for catalyzing the heme degradation were not conserved, suggesting that the ABM-like domain of PitA does not have heme degradation activity, but may possess the ability to bind with the heme molecule.

Based on the above discussion, we would like to propose a scenario for the biochemical function of PitA that is as follows: the protoheme molecules are synthesized in the ChdC domain, then migrate to the adjacent AMB-like domain, and are stored for supply to the heme-requiring components. Oxidative decarboxylation of the coproheme catalyzed by ChdC requires coexistence of a peroxide or flavin mononucleotide as the electron acceptor [10]. It has been reported that, when peroxide was added for an *in vitro* ChdC reaction, the total amount of coproheme that remained *plus* the protoheme generated in the reaction solution was lower than the expected value, indicating that the protoheme was degraded by the peroxidase reaction catalyzed by ChdC itself [10, 29]. Biosynthesis of protoheme catalyzed by PitA is, therefore, expected to proceed more efficiently if the protoheme generated in the ChdC domain is accepted immediately by the ABM-like domain to prevent the degradation. This proposal is speculative, and must be verified in future work.

## Conclusions

The phenotypic data on the *pitA* deletion variant presented in this paper is consistent with the suggestion of Dailey and Gerdes [8] that PitA might participate in the final step of the ChdC pathway in *H*. *volcanii*. Moreover, bioinformatic analysis of *H*. *volcanii* genome together with the phenotypic characterization of the *ahbD* deletion variant also suggests the presence of a functioning anaerobic heme biosynthetic process, by the Ahb pathway. Based on the present results, it is expected that in *H*. *volcanii*, and probably other halophilic archaea, heme can be produced under both aerobic and anaerobic conditions, through the ChdC and Ahb pathways, respectively. Here, it is notable that the *uroD* gene (encoding uroporphyrinogen decarboxylase), *cgoX* gene (coproporphyrinogen decarboxylase), and *cpfC* gene (coproporphyrin ferrochelatase) that are required for an aerobic ChdC pathway are absent in the *H*. *volcanii* genome (S1 Fig). This should not exclude the possibility of the existence of a yet unknown heme biosynthetic process in *H*. *volcanii* that produces a coproheme by a reaction process different from both of the ChdC and the Ahb pathways. Although PitA is expressed in the denitrifying cells as well as in the aerobic cells of *H*. *volcanii*, it seems to only be functional under aerobic growth conditions and therefore, in the aerobic heme biosynthesis pathway. Furthermore, the putative structural characteristics of PitA suggest that this protein is not only involved in heme biosynthesis but also in the binding of protoheme, which would hypothetically prevent its degradation. A detailed biochemical characterization of the purified PitA must be carried out to enlighten the hypothesis discussed above.

## Supporting information

S1 Fig Annotation of putative heme biosynthetic genes in the *H*. *volcanii* genome. The genes included in the protoporphyrin-dependent pathway for synthesis of protoheme from uroporphyrinogen III (Uropor’gen) was not identified in the *H*. *volcanii* genome except for the putative *pgdH1* gene (HVO_2669) encoding a menaquinone-dependent enzyme for anaerobic oxidation of protoporphyrinogen III (Protopor’gen). PgoX and CpfC are essential for the ChdC pathway, while the putative genes are absent in the *H*. *volcanii* genome. In contrast, the genes included in the anaerobic Ahb pathway, *hemX* (HVO_0077), *cysG* (HVO_2312), *nirDL* and *nirGH* (HVO_2227 and HVO_2313, respectively. Both are homologous to *ahbAB*), *AhbC* (HVO_1121), and *ahbD* (HVO_2144), were completely conserved. Grey and white arrows in the figure indicate the presence and absence of the putative genes encoding the corresponding enzymes, respectively. ALA, aminolevulinic acid; Copropor’gen, coproporphyrinigen III; Coproheme, Fe-coproporphyrin III; Protopor, protoporphyrin IX.
(TIF)

S2 Fig Confirmation of gene destruction. Genomic structure in the vicinity of *pitA* gene and *ahbD* gene was shown in (**A**), where oligonucleotide primers used for gene disruption and its confirmation are indicated. In (**B**), genotypes of the strains H26 (parent strain), P01 (pΔ*pitA* pop-in), and P02 (*pitA* gene deleted variant) were confirmed by PCR amplification of the corresponding genome region using two sets of primers, pitAUF/pitADR. Deletion of the *pitA* gene in the strain P02 was ascertained by PCR using a set of primers, pitAinF/pitADR (**C**). Destruction of the *ahbD* gene was also confirmed by amplification using two sets of primers, ahbDUF/ahbDDR and ahbDUF/ahbDinR, where strains A01 and A02 are the pΔ*ahbD* pop-in and *ahbD* gene deletion variants, respectively, as shown in (**C**) and (**D**). *Eco*T14I-digested λ phage genome (λEco) and *Hae*III-digested ΦX174 phage genome (ΦX) were used for the standard.
(TIF)

S3 Fig Effect of DMSO on the protoheme-dependent aerobic growth of the *H*. *volcanii* Δ*pitA* variant. The *pitA* mutant of *H*. *volcanii*, strain P02, was cultivated under aerobic condition. The strain P02 grew actively with 7.6 h t_d_ in the aerobic medium containing 5 μM protoheme and 35 mM DMSO by adding a protoheme/DMSO stock solution (closed triangles). The strain P02 grew more slowly (11.4 h t_d_) when the protoheme/Tris stock solution was used for supplementation of 5 μM protoheme to the medium (open triangles). Experiments were performed independently three times. Error bars represent S.E. The S.E. values were small, and therefore the error bars are sometimes masked by the symbols.
(TIF)

S4 Fig Absorption spectra of PitA in the visible-near ultraviolet region. Absorption spectra of the purified PitA in the oxidized state as isolated (solid line) and the dithionite-reduced state (dotted line) were measured. The spectrum of the reduced state PitA in complex with carbon monoxide (CO) shown by a dash-dot line was measured after the reduced sample was gently bubbled with pure CO gas.
(TIF)

S5 Fig Sequence alignment of the ABM-like domain of PitA. Amino acid sequence of the ABM-like domain of the *H*. *volcanii* PitA (corresponding to the fraction II shown in Fig 3B) was aligned with those of heme-containing ABM enzymes harboring heme-degradation activity. Typical ferredoxin-like βαββαβ-foldings of *S*. *aureus* IsdG (PDB ID code: 1XBW) and IsdI (3LGN), *Mycobacterium tuberculosis* MhuD (4NL5), and *Bacillus subtilis* HmoB (3TVZ) have already been solved by crystal structural analysis [37–39]. Secondary structures, two or three helices (grey cylindrical) and four sheets (dark grey arrows), conserved among the four enzymes are shown at the top of the corresponding bolded sequences. The three residues, Asn^7^, Trp^67^, and His^77^ (*S*. *aureus* IsdG numbering), that are essential for the heme-degradation activity are boxed [37]. The secondary structure of the ABM-like domain of the PitA predicted by using PSIPRED (http://bioinf.cs.ucl.ac.uk/psipred/) is indicated at the bottom of the corresponding italicized sequences. Only His^444^ (*H*. *volcanii* PitA numbering), the putative proximal ligand of the protoheme, was conserved in the PitA, while Asn and Trp were replaced by Thr^333^ and Val^434^, respectively. An inserted region with 41 residues (386^th^–426^th^) including a putative β-sheet structure is indicated by lower case letters between the β3 and the α2. Additional α-helices were predicted at the C-terminal of PitA.
(TIF)

S1 Table Oligonucleotide primers used for PCR amplification. (DOCX)

S1 Methods Large-scale cultivation, preparation of the *ahbD* gene deletion variant, purification of PitA. (DOCX)
