# Supplementary figures and images for: Growth phenotype analysis of heme synthetic enzymes in a halophilic archaeon, Haloferax volcanii
Source: PLoS One. 2017 Dec 28;12(12):e0189913. doi: 10.1371/journal.pone.0189913 (PMC5746218; doi:10.1371/journal.pone.0189913)

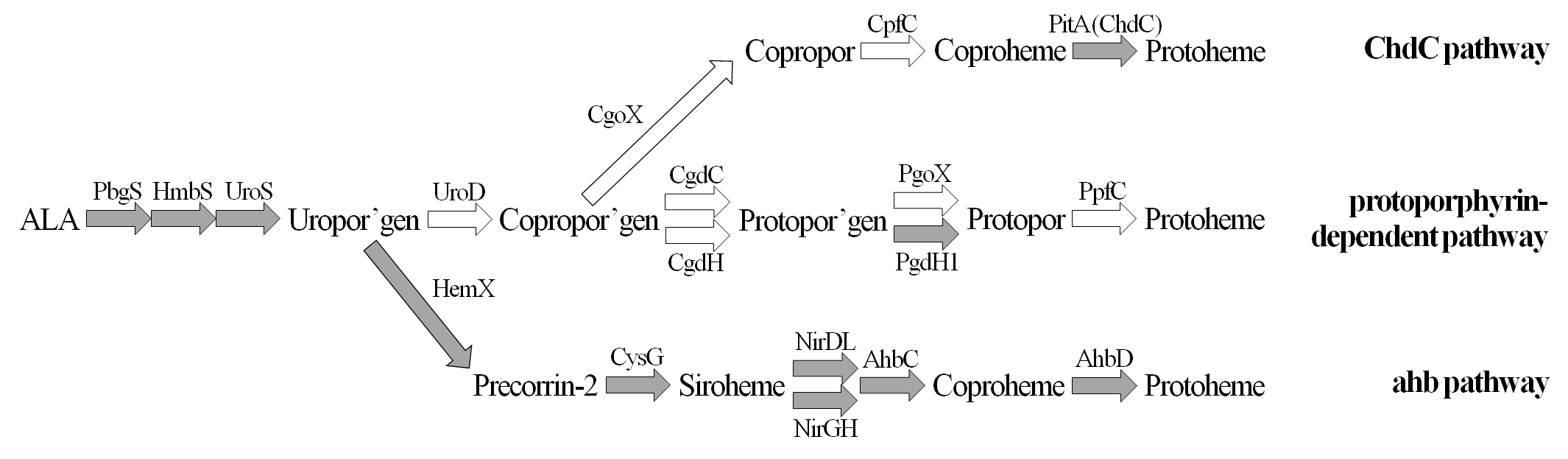

Supplement: S1 Fig — The genes included in the protoporphyrin-dependent pathway for synthesis of protoheme from uroporphyrinogen III (Uropor’gen) was not identified in the H. volcanii genome except for the putative pgdH1 gene (HVO_2669) encoding a menaquinone-dependent enzyme for anaerobic oxidation of protoporphyrinogen III (Protopor’gen). PgoX and CpfC are essential for the ChdC pathway, while the putative genes are absent in the H. volcanii genome. In contrast, the genes included in the anaerobic Ahb pathway, hemX (HVO_0077), cysG (HVO_2312), nirDL and nirGH (HVO_2227 and HVO_2313, respectively. Both are homologous to ahbAB), AhbC (HVO_1121), and ahbD (HVO_2144), were completely conserved. Grey and white arrows in the figure indicate the presence and absence of the putative genes encoding the corresponding enzymes, respectively. ALA, aminolevulinic acid; Copropor’gen, coproporphyrinigen III; Coproheme, Fe-coproporphyrin III; Protopor, protoporphyrin IX. (TIF) [file pone.0189913.s001.tif]

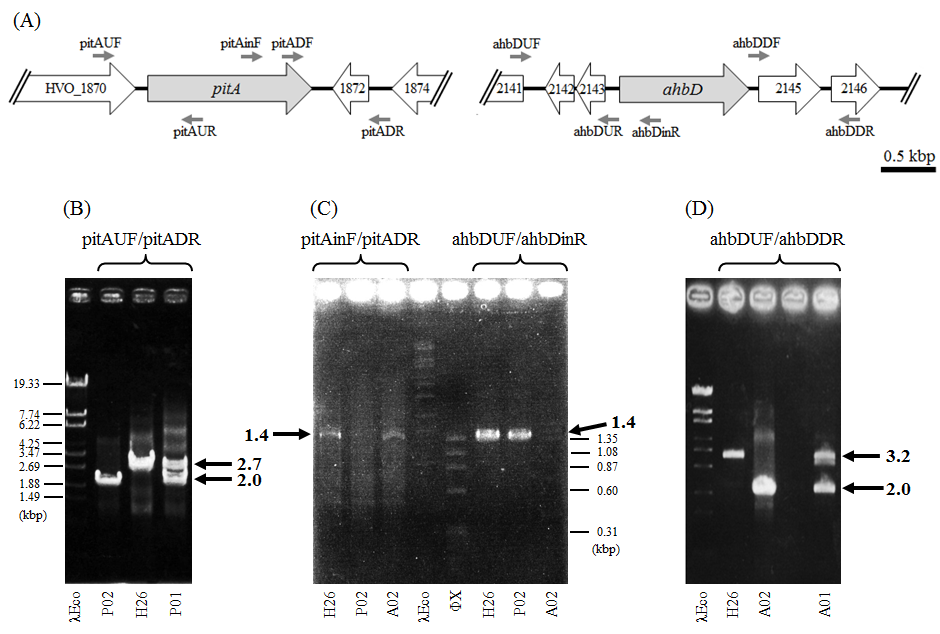

Supplement: S2 Fig — Genomic structure in the vicinity of pitA gene and ahbD gene was shown in (A), where oligonucleotide primers used for gene disruption and its confirmation are indicated. In (B), genotypes of the strains H26 (parent strain), P01 (pΔpitA pop-in), and P02 (pitA gene deleted variant) were confirmed by PCR amplification of the corresponding genome region using two sets of primers, pitAUF/pitADR. Deletion of the pitA gene in the strain P02 was ascertained by PCR using a set of primers, pitAinF/pitADR (C). Destruction of the ahbD gene was also confirmed by amplification using two sets of primers, ahbDUF/ahbDDR and ahbDUF/ahbDinR, where strains A01 and A02 are the pΔahbD pop-in and ahbD gene deletion variants, respectively, as shown in (C) and (D). EcoT14I-digested λ phage genome (λEco) and HaeIII-digested ΦX174 phage genome (ΦX) were used for the standard. (TIF) [file pone.0189913.s002.tif]

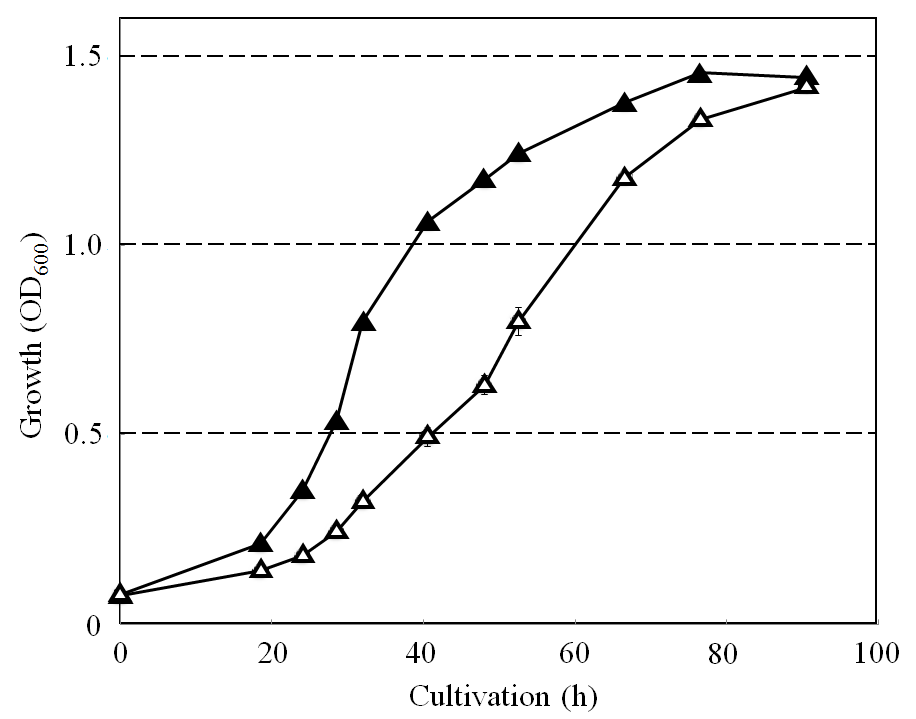

Supplement: S3 Fig — The pitA mutant of H. volcanii, strain P02, was cultivated under aerobic condition. The strain P02 grew actively with 7.6 h td in the aerobic medium containing 5 μM protoheme and 35 mM DMSO by adding a protoheme/DMSO stock solution (closed triangles). The strain P02 grew more slowly (11.4 h td) when the protoheme/Tris stock solution was used for supplementation of 5 μM protoheme to the medium (open triangles). Experiments were performed independently three times. Error bars represent S.E. The S.E. values were small, and therefore the error bars are sometimes masked by the symbols. (TIF) [file pone.0189913.s003.tif]

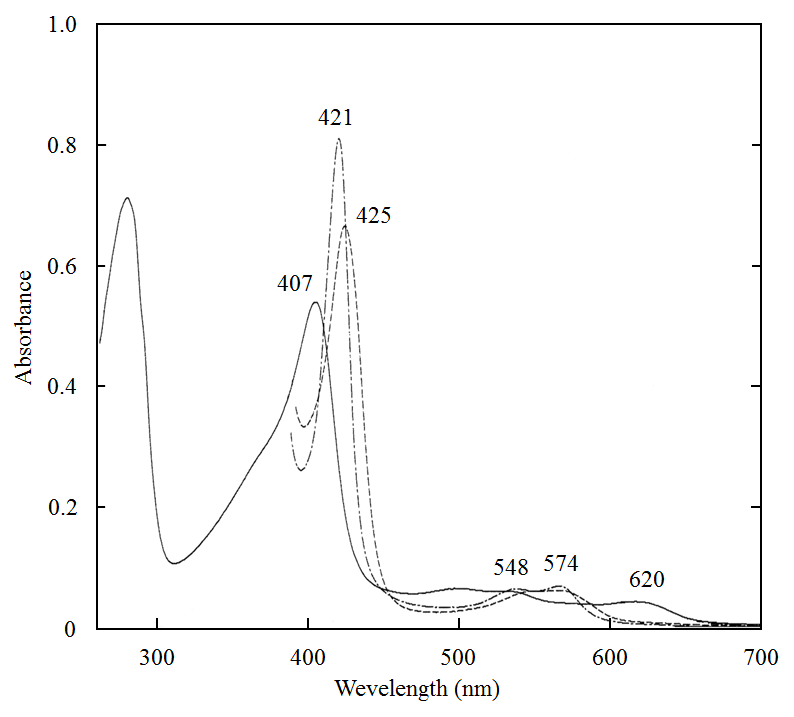

Supplement: S4 Fig — Absorption spectra of the purified PitA in the oxidized state as isolated (solid line) and the dithionite-reduced state (dotted line) were measured. The spectrum of the reduced state PitA in complex with carbon monoxide (CO) shown by a dash-dot line was measured after the reduced sample was gently bubbled with pure CO gas. (TIF) [file pone.0189913.s004.tif]

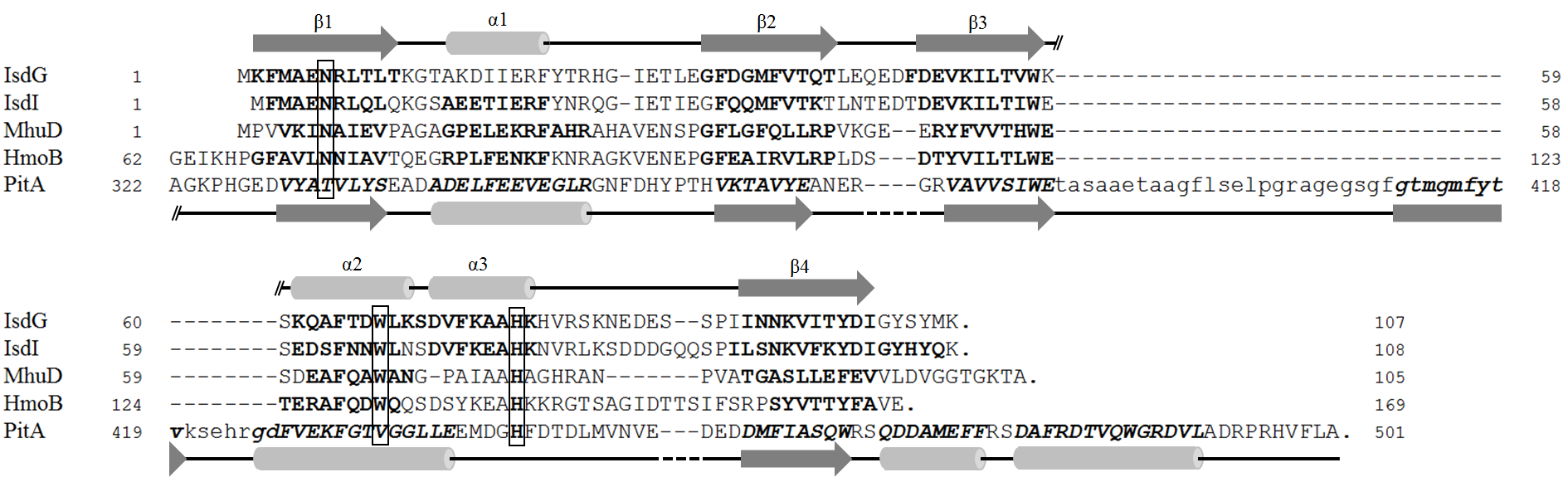

Supplement: S5 Fig — Amino acid sequence of the ABM-like domain of the H. volcanii PitA (corresponding to the fraction II shown in Fig 3B) was aligned with those of heme-containing ABM enzymes harboring heme-degradation activity. Typical ferredoxin-like βαββαβ-foldings of S. aureus IsdG (PDB ID code: 1XBW) and IsdI (3LGN), Mycobacterium tuberculosis MhuD (4NL5), and Bacillus subtilis HmoB (3TVZ) have already been solved by crystal structural analysis [37–39]. Secondary structures, two or three helices (grey cylindrical) and four sheets (dark grey arrows), conserved among the four enzymes are shown at the top of the corresponding bolded sequences. The three residues, Asn7, Trp67, and His77 (S. aureus IsdG numbering), that are essential for the heme-degradation activity are boxed [37]. The secondary structure of the ABM-like domain of the PitA predicted by using PSIPRED (http://bioinf.cs.ucl.ac.uk/psipred/) is indicated at the bottom of the corresponding italicized sequences. Only His444 (H. volcanii PitA numbering), the putative proximal ligand of the protoheme, was conserved in the PitA, while Asn and Trp were replaced by Thr333 and Val434, respectively. An inserted region with 41 residues (386th–426th) including a putative β-sheet structure is indicated by lower case letters between the β3 and the α2. Additional α-helices were predicted at the C-terminal of PitA. (TIF) [file pone.0189913.s005.tif]
