# Supplementary material for: Growth phenotype analysis of heme synthetic enzymes in a halophilic archaeon, Haloferax volcanii
Source: PLoS One. 2017 Dec 28;12(12):e0189913. doi: 10.1371/journal.pone.0189913 (PMC5746218; doi:10.1371/journal.pone.0189913)
Supplement: S1 Table — (DOCX) [file pone.0189913.s006.docx]

**S1 Table.** **Oligonucleotide primers used for PCR amplification.**

| **Name** | **Nucleotide sequence (5’-3’)** | **Remarks** |
| --- | --- | --- |
| **pitAUF** | GAG CTC CGC CCC TCC AGC TCG TCT ACG | inserted *Sac*I underlined |
| **pitAUR** | GGA TCC TCG TCG AAC GAG AGG TCG TAC | inserted *Bam*HI underlined |
| **pitADF** | GGA TCC GCC GGC GAG GGG TCC GGC TTC | inserted *Bam*HI underlined |
| **pitADR** | CTG CAG GAC CGC CGG AAA AAG GAC CCG | inserted *Pst*I underlined |
| **pitAinF** | CAC CAC CAT CAC GAC GAC GGC |  |
| **ahbDUF** | GGA TCC GTC GAA CCC TGT TGT GTC GG | inserted *Bam*HI underlined |
| **ahbDUR** | GGC TCT TGT CGT GCG GTT CG |  |
| **ahbDDF** | CGC CTC GAG AAG TGG CAG CG |  |
| **ahbDDR** | GGA TCC CGC CGC GGA GTC GGG AGG | inserted *Bam*HI underlined |
| **ahbDinR** | GGC GTG AGA GTC ATC CTG AG |  |
