# Supplementary material for: Growth phenotype analysis of heme synthetic enzymes in a halophilic archaeon, Haloferax volcanii
Source: PLoS One. 2017 Dec 28;12(12):e0189913. doi: 10.1371/journal.pone.0189913 (PMC5746218; doi:10.1371/journal.pone.0189913)
Supplement: S1 Methods — (DOCX) [file pone.0189913.s007.docx]

**Methods**

**Large-scale cultivation**

Large-scale cultivation of strain H26 in the aerobic condition was performed as follows. A 500 mL flask was filled with 200 mL Hv medium and capped by a silicone sponge closure. After inoculation of 1/100 volume of the aerobic culture of the cells, the flask was shaken at 120 rpm at 37°C. The cells in the late-exponential phase (OD_600_ = ~1.5) were harvested by centrifugation at 7,000 × g for 30 min using a model Himac CR-21 centrifuge (Hitachi-Koki Co., Ltd., Tokyo, Japan). The cell pellet obtained was stored at -30°C until used for the purification of PitA. Large-scale cultivation in the denitrifying condition was also performed as follows. A 500 mL flask was filled with the Hv medium containing 50 mM KNO_3_ and sealed with aluminum foil. After inoculation, static cultivation was carried out at 37°C. The cells in the late-exponential growth phase (OD_600_ = ~0.7) were harvested centrifugally, and used as the starting material for the purification of PitA as described below.

**Preparation of the *ahbD* gene deletion variant**

The *ahbD* gene was also destroyed according to an experimental procedure that was similar to the preparation of the *pitA* gene deletion variant. The 3.2 kbp fragment including upstream (1.0 kbp) and downstream (1.0 kbp) regions of the *ahbD* gene was amplified using a set of ahbDUF/ahbDDR oligonucleotide primers. Sequences of the primers are shown in S1 Table. The amplified fragment was cloned into a pCR-blunt II TOPO vector, yielding pCR*ahbD*. Next, an inverse-PCR amplification was carried out using the pCR*ahbD* plasmid as the template and a set of ahbDUR/ahbDDF primers to remove the total region of the *ahbD* gene (1.2 kbp). The product was purified, and then subjected to self-ligation, yielding pCRΔ*ahbD*. The inserted fragment of 2.0 kbp nucleotides that was extracted by *Bam*HI digestion was cloned into the same restriction site of pTA131, generating p∆*ahbD*. The plasmid was introduced into *H. volcanii* strain H26 for integration into the genome DNA. Colonies of the pop-in strain, designated A01 (genotype: ∆*pyrE2* *ahbD*^+^ [*pyrE2* ∆*ahbD*]), appeared on the Hv-Ca agar medium. Strain A01 was pre-cultivated under aerobic conditions in the Hv-Ca medium. Cultivated A01 cells were streaked on the Hv-Ca agar medium supplemented with 50 μM 5’-FOA and 50 μM uracil for a second homologous recombination event. The agar medium was incubated at 37°C. The genotype of the colonies that appeared on the agar medium was analyzed by PCR amplification using the ahbDUF/ahbDDR primer set (S2 Fig). The *ahbD* gene deletion variant (genotype: ∆*ahbD* ∆*pyrE2*) thus obtained was designated A02.

**Purification of PitA**

PitA was purified from *H. volcanii* H26 cells cultivated under aerobic or denitrifying conditions according to Bab-Dinitz *et al*. [18] with some modifications. Aerobically cultivated cells of *H. volcanii* strain H26, which were harvested from 10 L Hv medium, were suspended in 100 mL of 20 mM sodium phosphate buffer (pH 7.2) containing 2 M NaCl (buffer A). The suspension was disrupted using a VP-30S supersonic oscillator (Taitec), then centrifuged at 18,000 × g for 20 min to remove unbroken cells and debris. The cell-free extract was centrifuged at 140,000 × g for 1 h by using an ultracentrifuge (Optima L-90K, Beckman Coulter Inc., Brea, CA), then the supernatant thus obtained was used as the starting material for the purification of PitA. The supernatant was dialyzed against the buffer A at 4°C for 12 h. The resulting solution was subjected to a Ni^2+^-chelating Sepharose (Chelating Sepharose Fast Flow, Amersham Bioscience AB, Uppsala, Sweden) column (2.5 cm × 10 cm) that had been equilibrated with buffer A. After loading the solution, the column was washed with 70 ml of buffer A, and then with 100 ml of buffer A containing 0.5 M sodium glutamate. The PitA adsorbed on the column was eluted by a linear gradient generated from 100 mL each of buffer A and buffer A containing 0.15 M imidazole. The partially purified PitA obtained was mixed with fine crystals of (NH_4_)_2_SO_4_ to reach 60% saturation. The solution was loaded onto a Sepharose CL-4B (Pharmacia) column (2.5 cm × 10 cm) that had been equilibrated with buffer A which was 60% saturated with (NH_4_)_2_SO_4_. After washing the column with 100 mL of the same solution, the PitA adsorbed on the column was eluted with a linear gradient generated by 100 mL each of the same solution and buffer A. Eluates whose ratios of absorbance at 406 nm to that at 280 nm (A_406_/A_280_) were higher than 0.5 were collected. The preparation obtained was concentrated using a Microcon YM-100 filter (Millipore Co., Bedford, MA), then was loaded onto a column (2 cm × 120 cm) of Sephacryl S-200 (Pharmacia) that had been equilibrated with buffer A. The eluates whose A_406_/A_280_ values were higher than 0.8 were collected, concentrated using the Microcon YM-100, and used as the purified preparation for the experiments. PitA was also purified from the denitrifying cells of *H. volcanii* that was harvested in the late-exponential growth phase according to the above mentioned procedure.
